# Disseminated Intravascular Coagulation in a Patient With Metastatic Adenocarcinoma: A Case of Insidious Progression and Hematologic Complications

**DOI:** 10.7759/cureus.95839

**Published:** 2025-10-31

**Authors:** Christine L Zickler, Matthew Maguire, Melanie Molina, Michelle Braha, Guillermo Izquierdo-Pretel

**Affiliations:** 1 Internal Medicine, Florida International University, Herbert Wertheim College of Medicine, Miami, USA; 2 Internal Medicine, Florida International University, Miami, USA

**Keywords:** adenocarcinoma, coagulation, dic, disseminated intravascular coagulation, hematologic disorder

## Abstract

Disseminated intravascular coagulation (DIC) is a severe condition characterized by systemic activation of coagulation, resulting in the formation of microthrombi and consumption of clotting factors, leading to a paradoxical risk of both thrombosis and bleeding. Early diagnosis is crucial to initiating appropriate treatment before the condition worsens, as DIC is often associated with a poor prognosis once clinical signs appear. This case report presents a 60-year-old female patient with metastatic adenocarcinoma, initially diagnosed two years prior, who developed acute DIC shortly before her death. Despite an initial presentation of intractable back pain, nausea, and generalized weakness, laboratory findings demonstrated hemolytic anemia and abnormal coagulation, leading to the diagnosis of consumptive coagulopathy secondary to her advanced malignancy. The patient’s declining condition was accompanied by worsening laboratory parameters, including elevated prothrombin time, international normalized ratio (INR), and fibrin degradation products, highlighting the insidious progression of DIC in cancer patients, particularly in the context of adenocarcinoma. Although treated with supportive care, including blood transfusions and withdrawal of anticoagulation, she ultimately succumbed to cardiopulmonary arrest. This case emphasizes the importance of early recognition of DIC in oncology patients, especially those with advanced-stage malignancies, as it often manifests in a less aggressive yet equally fatal form compared to DIC seen in conditions such as sepsis or trauma. Additionally, it emphasizes the need for vigilant laboratory monitoring in critically ill cancer patients to facilitate timely intervention.

## Introduction

Disseminated intravascular coagulation (DIC) is a life-threatening condition characterized by systemic activation of the coagulation cascade, leading to both thrombosis and hemorrhage. It arises when the body’s clotting mechanisms are overwhelmed, resulting in widespread formation of microthrombi and subsequent consumption of platelets and coagulation factors. This dual threat of excessive clotting and bleeding can severely compromise organ function and patient survival. DIC is often seen as a complication of conditions such as sepsis, trauma, or malignancy, with its manifestation influenced by the underlying pathology [1].

In cancer patients, particularly those with solid tumors such as adenocarcinoma, DIC typically evolves in a more chronic, insidious manner compared with the acute, fulminant course observed in sepsis or trauma. Although its incidence is lower in malignancy (5-10%) than in sepsis or trauma (30-50%), it remains clinically significant [2]. Hallmark features include thrombocytopenia, ecchymosis, schistocytes on peripheral smear, and prolonged international normalized ratio/prothrombin time (INR/PT) [3]. Tumor-mediated prothrombotic activity further predisposes patients to DIC through mechanisms such as tissue factor expression, cytokine release, and endothelial injury from chemotherapy. Despite its slower onset in oncology settings, DIC remains a major contributor to morbidity and mortality, underscoring the importance of early recognition and timely management [4].

Diagnosis of DIC is largely clinical, based on a combination of laboratory findings such as prolonged activated partial thromboplastin time (APTT), PT, elevated D-dimer, and low platelet count. While fibrinogen levels are useful markers, they may remain normal due to their role as an acute-phase reactant, necessitating serial measurements for a more accurate assessment [5].

This case report describes a 60-year-old female patient with metastatic adenocarcinoma who developed acute DIC during her hospital admission. Her case highlights the complexity of diagnosing DIC in cancer patients, the challenges of managing coagulopathy in the context of malignancy, and the importance of vigilant monitoring for early signs of hematologic abnormalities in high-risk individuals. Notably, her presentation is unusual in that acute DIC occurred in the terminal phase of metastatic adenocarcinoma, suggesting that DIC may at times represent a manifestation of end-stage disease.

## Case presentation

Clinical findings

The patient is a 60-year-old female with metastatic adenocarcinoma who experienced acute DIC during her hospitalization. She was alert and oriented ×3, with gross motor and sensory function intact on admission. On physical examination, the patient appeared frail and ill, with prominent scleral icterus. There was significant tenderness to palpation along the vertebral column and sternum, with limited flexion and extension of the spine due to pain. Abdominal exam revealed distention without rebound or guarding. There was no evidence of active bleeding or overt sepsis. Inspection of the skin revealed diffuse ecchymosis, more pronounced on the bilateral lower extremities, with more ecchymosis on the left than the right. Ecchymosis was also present on the bilateral upper extremities, notably at current and previous IV insertion sites. The bruising was noted to be spontaneous and progressive over the course of admission. The respiratory, cardiovascular, and abdominal examinations were otherwise unremarkable. The patient reported additional symptoms, including fatigue, shortness of breath, pain with deep inspiration, constipation, neuropathy, and easy bruising. The remainder of the review of systems was otherwise unremarkable.

History

Two years earlier, she was diagnosed with adenocarcinoma of unknown primary, which progressed to peritoneal carcinomatosis with widespread metastases involving the uterus, bones, and lungs. Her prior systemic therapies included capecitabine and bevacizumab, discontinued two months before admission. She was followed by palliative care, managed with opioids and celecoxib, and underwent thoracocentesis without cytology one month prior to admission.

Chief concerns

Her presenting symptoms included progressive back pain, nausea, and worsening inability to tolerate oral intake, which had advanced from solids to liquids.

Diagnostic assessment

**Figure 1:**
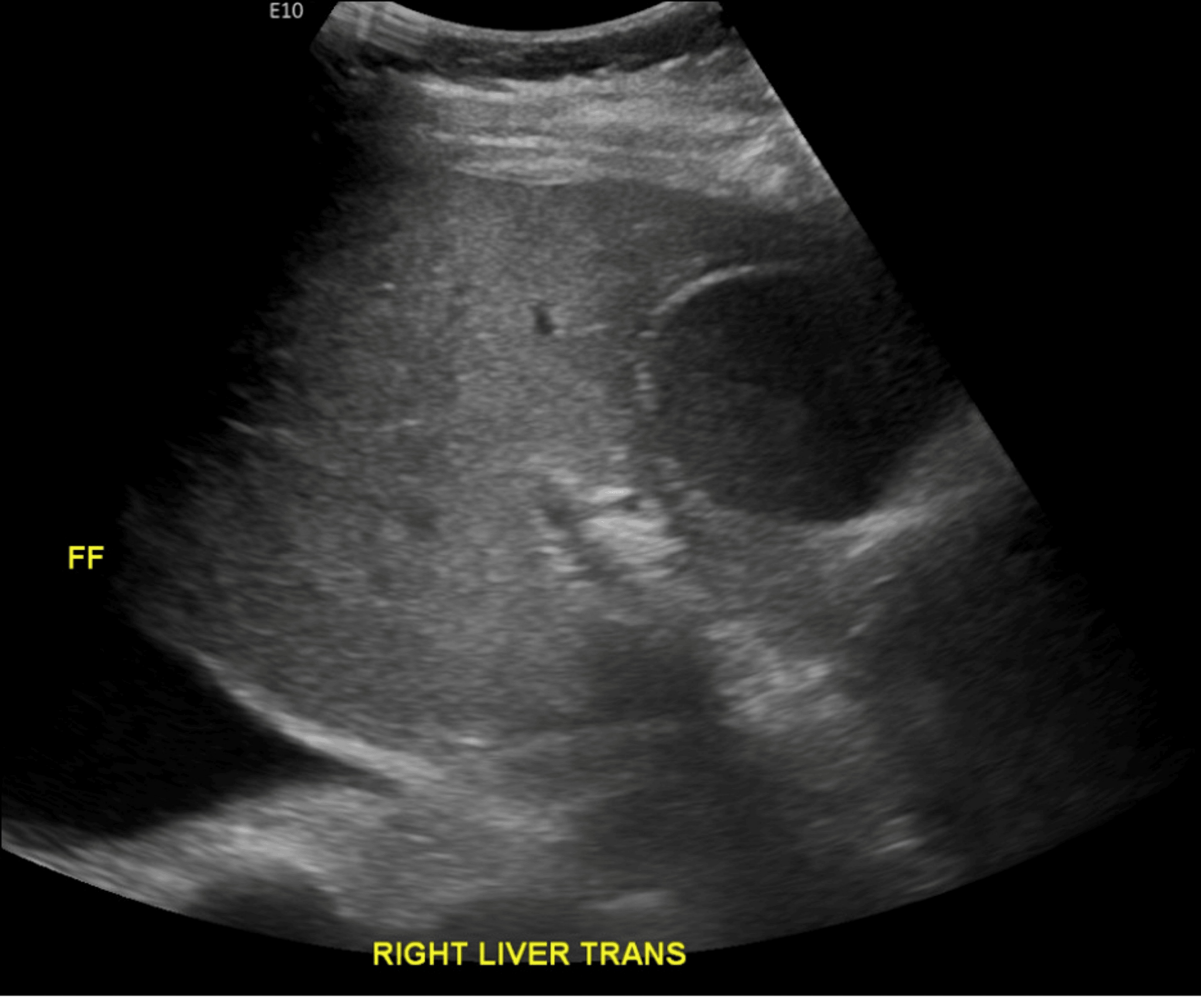
US abdomen US: ultrasound; FF: Free fluid noted Sludge is seen within the gallbladder. No discrete shadowing stones are seen. Thickened gallbladder wall measuring 4 mm. No pericholecystic fluid. Positive Murphy sign

**Figure 2:**
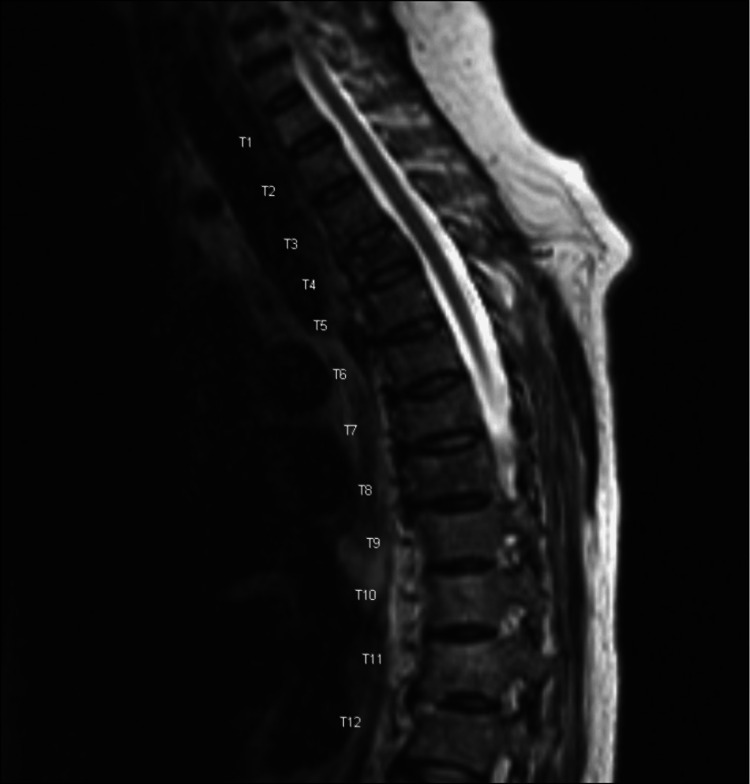
MR thoracic w/o contrast MRI: magnetic resonance imaging Pathologic compression fracture of T4 with 25-50% height loss with mild posterior bulging of the vertebral body without evidence of significant central canal narrowing. Pathologic compression fracture of T7 with less than 25% height loss

Abdominal ultrasound showed distention of the gallbladder with sludge, as well as re-demonstrated abdominopelvic ascites (Figure 1). Computed tomography scan of the abdomen and pelvis revealed malignant ascites, gallbladder distention, small bilateral pleural effusions, and progressive osseous metastatic disease, including worsening lesions at T4 and T7, along with multiple pathologic fractures (Figure 2). A hepatobiliary scan showed visualization of the liver, bile ducts, and bowel within the expected period of time. There was also visualization of the gallbladder starting at approximately 20 minutes and visualization of the bowel within 30 minutes. The scan showed no scintigraphic evidence of obstruction of the cystic duct or common bile duct.

Laboratory results from days 1 through 3 are shown in Tables 1-3. Daily metabolic panel and complete blood counts can be seen in Table 4. Of note, on day 3, the peripheral blood smear showed light smudge cells, schistocytes, and moderate acanthocytosis.

**Table 1:** Day 1 laboratory results

Lab parameters (units)	Patient value	Reference range
Lactic acid (mmol/L)	2	0.5-2.2
Lipase (U/L)	61	10-140

**Table 2:** Day 2 laboratory results

Lab parameters (units)	Patient value	Reference range
Direct bilirubin (mg/dL)	1.3 (H)	0-0.3
Amylase (U/L)	36	30-110
Magnesium (mg/dL)	1.7	1.7-2.2

**Table 3:** Day 3 laboratory results INR: international normalized ratio; TIBC: total iron-binding capacity; LDH: lactate dehydrogenase

Lab parameters (units)	Patient value	Reference range
Prothrombin time (seconds)	31.5 (H)	10-13
INR	2.97 (H)	0.8-1.2
Fibrinogen (mg/dL)	196 (L)	200-400
Magnesium (mg/dL)	1.6 (L)	1.7-2.2
TIBC (µg/dL)	237 (L)	250-450
Ferritin (ng/mL)	4734 (H)	13-150
Vitamin B12 (pg/mL)	3890 (H)	200-900
Haptoglobin (mg/dL)	<10 (L)	30-200
Iron (µg/dL)	204 (H)	50-170
LDH (U/L)	1714 (H)	140-280
Reticulocyte count (%)	8.2 (H)	0.5-2.5
Absolute reticulocyte count (M/µL)	0.18 (H)	0.025-0.075
Reticulocyte hemoglobin (pg)	29.2	28-35
Immature reticulocyte fraction (%)	39.5% (H)	11-34

**Table 4:** Daily metabolic panel and complete blood count BUN: blood urea nitrogen; AST: aspartate aminotransferase; ALT: alanine aminotransferase; MCV: mean corpuscular volume; MCH: mean corpuscular hemoglobin; MCHC: mean corpuscular hemoglobin concentration; RDW: red cell distribution width

	Day 1	Day 2	Day 3	Reference range
Glucose (mg/dL)	137 (H)	123 (H)	143 (H)	70-99
Sodium (mmol/L)	130 (L)	134 (L)	136 (L)	135-145
Potassium (mmol/L)	4.8	4.5	4.1	3.5-5.0
Chloride (mmol/L)	95 (L)	99	104	96-106
CO2 (mmol/L)	30	32 (H)	29	22-29
Anion gap (mmol/L)	5 (L)	3 (L)	3 (L)	8-16
Osmolality calculated (mOsm/kg)	267 (L)	271 (L)	277	275-295
BUN (mg/dL)	26 (H)	18 (H)	20	6-20
Creatinine (mg/dL)	0.63	0.50 (L)	0.40 (L)	0.6-1.3
Calcium (mg/dL)	10.7 (H)	10.1	9.9	8.5-10.5
Total protein (g/dL)	6.7	5.6 (L)	5.5 (L)	6.4-8.3
Albumin (g/dL)	4	3.2 (L)	3.0 (L)	3.5-5
Total bilirubin (mg/dL)	4.5 (H)	4.6 (H)	5.4 (H)	0.1-1.2
AST (U/L)	80 (H)	80 (H)	89 (H)	10-40
ALT (U/L)	34	26	24	7-56
Alkaline phosphatase (U/L)	371 (H)	335 (H)	308 (H)	44-147
WBC (thousdand/µL)	6.1	5.7	5.5	4.5-11
RBC (thousdand/µL)	2.86 (L)	2.54 (L)	2.16 (L)	4.1-5.9
Hemoglobin (g/dL)	8.1 (L)	7.2 (L)	6.2 (L)	12.1-15.1
Hematocrit (%)	25.3 (L)	22.8 (L)	19.4 (L)	36-44
MCV (fL)	88.5	89.8	89.8	80-100
MCH (pg)	28.3	28.3	28.7	27-33
MCHC (g/dL)	32	31.5 (L)	32	32-36
RDW (%)	18.6 (H)	19.5 (H)	20.3 (H)	11.5-14.5
Platelets (thousand/µL)	61 (L)	60 (L)	49 (L)	150-450

Therapeutic intervention

The patient was started on metoclopramide and ondansetron as needed for nausea and vomiting, with symptomatic improvement by hospital day 3. By this time, she was able to tolerate oral intake with nutritional supplementation (Ensure Plus). Following the identification of hemolytic anemia, her anticoagulation with apixaban, previously prescribed for a deep vein thrombosis, was discontinued. She subsequently received one unit of packed red blood cells.

Management was consistent with guideline-based recommendations, emphasizing supportive transfusion, withdrawal of anticoagulation, and treatment of the underlying malignancy [6]. Vitamin K and corticosteroids were not administered, as there was no evidence of vitamin K deficiency or immune-mediated hemolysis.

Follow-up and outcomes

On day 3, the hematology/oncology team assessed the patient and diagnosed her with consumptive coagulopathy secondary to advanced malignancy. Although her fibrinogen levels were within normal limits, the insidious nature of DIC in cancer patients was recognized. Daily PT/INR, PTT, fibrinogen, and lactate dehydrogenase (LDH) were recommended for monitoring, and a Coombs test was ordered to rule out autoimmune hemolytic anemia (AIHA). Goals of care were discussed with the family that afternoon, during which the patient’s code status was changed to do not resuscitate and do not intubate (DNR/DNI). Later that evening, the patient was found unresponsive by nursing staff, and a rapid response was initiated, during which she was initially found to have a pulse. She subsequently lost pulses, briefly achieved return of spontaneous circulation (ROSC), and then re-arrested. Consistent with the updated goals of care, no further resuscitative efforts were pursued. Her condition continued to deteriorate, and she ultimately experienced cardiopulmonary arrest, passing away approximately one hour later.

## Discussion

A limitation of this case is the absence of an autopsy, which prevents definitive exclusion of alternative causes of cardiopulmonary arrest, such as pulmonary embolism. Nonetheless, a strength lies in the consistent clinical monitoring and regular laboratory testing throughout admission. This enabled prompt recognition of hemolytic anemia and close tracking of disease progression. The diagnosis of DIC was supported by a downward platelet trend (61k to 49k), markedly prolonged INR (2.97), elevated LDH (1714 U/L), and the presence of schistocytes on peripheral smear. A negative Coombs test further supported microangiopathic hemolytic anemia rather than autoimmune hemolysis.

Differential diagnoses such as sepsis, trauma, or liver dysfunction were considered. However, the absence of infection, lack of recent trauma, and only mild hepatic impairment favored malignancy-associated DIC as the most likely diagnosis. The timing of this patient’s acute DIC, arising within days of death despite supportive care, suggests that in advanced adenocarcinoma, DIC may represent a terminal manifestation with grave prognostic significance.

Cancer induces a prothrombotic state through mechanisms such as tissue factor-initiated coagulation, cytokine-mediated anticoagulation dysfunction, and endothelial injury from chemotherapy or radiation [7]. In cancer patients, DIC often presents less aggressively compared to cases associated with sepsis or trauma. While it may progress slowly and asymptomatically, it can still lead to significant clinical events. Incidence varies by malignancy type, with acute leukemias demonstrating the highest rates, while solid tumors such as adenocarcinomas are more often associated with thrombotic forms of DIC [2].

Managing DIC in malignancy presents significant challenges. As highlighted in prior case reports, Rosen et al. described a 55-year-old man with hepatic angiosarcoma complicated by DIC, presenting with rectal bleeding, hepatomegaly, and coagulopathy. Treatment included antifibrinolytics, heparin, romiplostim, and chemotherapy aimed at tumor control [8]. Similarly, Sohal et al. reported the difficulties in achieving long-term stabilization of DIC in malignancy, emphasizing that chemotherapy should be initiated promptly once coagulopathy is stabilized [9]. Giszas et al. further described the importance of frequent monitoring and targeted therapy, noting that delayed intervention can lead to rapid clinical decline [10]. Ultimately, our case underscores the importance of aligning diagnostic vigilance and supportive management with patient goals of care, particularly when DIC arises in the terminal phase of advanced adenocarcinoma.

## Conclusions

This case highlights the importance of early recognition and management of DIC in patients with advanced malignancy. Although DIC in cancer often presents and progresses insidiously, it remains a major source of morbidity and mortality. In this patient, the onset of acute DIC likely signaled terminal decline in the setting of metastatic adenocarcinoma. Close laboratory monitoring can facilitate timely recognition, but the prognosis in advanced disease remains poor. In such cases, aggressive interventions may not align with patient goals of care, making supportive management the most appropriate approach. Ultimately, heightened awareness and continued research are needed to improve diagnostic strategies and optimize management of DIC in oncology patients.
